# Plastic surgery and adjuvant chemoradiotherapy in the management of advanced external auditory canal cancer

**DOI:** 10.3332/ecancer.2022.1432

**Published:** 2022-07-20

**Authors:** Dang Nguyen Van, Thanh Lap Bui, Hoan Pham Van, Minh The Dao, To Ta Van

**Affiliations:** 1 Department of Oncology, Hanoi Medical University, 01 Ton That Tung Street, Dong Da District, Hanoi 100000, Vietnam; 2 Department of Head and Neck Radiation Oncology, Vietnam National Cancer Hospital, Hanoi 100000, Vietnam; 3 Department of Medical Oncology 1, Vietnam National Cancer Hospital, Hanoi 100000, Vietnam; 4 Center of Pathology and Molecular biology, Vietnam National Cancer Hospital, Hanoi 100000, Vietnam; a https://orcid.org/0000-0002-3370-478X

**Keywords:** squamous cell carcinoma, external auditory canal, chemoradiotherapy, advanced stages

## Abstract

**Background:**

Carcinoma of the external auditory canal is a rare malignancy that originates in the external auditory canal and has a tendency to spread to adjacent structures such as the periorbital soft tissues, parotid gland, temporomandibular joint and mastoid. It is difficult to determine the primary tumour from the external ear canal or from adjacent structures. In addition, the lack of a unified classification system, along with the rarity of the disease, is a major obstacle in providing specific treatment guidelines for this disease.

**Case presentation:**

In this report, we describe a clinical case of a 59-year-old male patient who was admitted to the hospital because of ear pain and discharge from the outer ear canal with swelling in the right temporal region. The initial diagnosis is highly suggestive of an advanced external auditory canal malignancy with involvement of the parotid gland. The patient underwent surgery to remove the entire tumour, neck lymph nodes, followed by plastic surgery. The patient then received adjuvant chemoradiotherapy. There was no recurrence of cancer for 28 months, to date, after completing treatment.

**Conclusions:**

Extensive tumour resection combined with reconstruction and adjuvant chemoradiotherapy is a treatment strategy with good outcomes for patients with advanced squamous cell carcinoma of the external ear canal.

## Background

External ear canal cancer is a relatively rare disease, accounting for less than 0.2% of head and neck cancers and with an annual incidence of 1–6/1,000,000 people [1]. Most people are between the ages of 50 and 60 [2]. Patients frequently present with ear discomfort, tinnitus and unresponsive chronic ear infections. These signs are frequently missed or misdiagnosed as symptoms of other diseases, and patients frequently present to the hospital with advanced disease [3–5]. Endoscopy, along with biopsy, as well as other imaging technologies, is used to provide a clear diagnosis and evaluate the degree of the disease. Due to the rarity of these disorders, the American Joint Committee on Cancer (AJCC) and Union for International Cancer Control (UICC) do not yet have a classification system for them. Numerous categorisation systems are used in clinical practice; the most widely used is the Pittsburgh classification, which was proposed by Arriaga *et al* [6] in 1990 and refined by Moody *et al* [7] in 2000. Five-year survival is projected to be 85%–100% for stage I or stage II disease, 50%–68.8% for stage III disease and just 19.6%–30% for stage IV cancer [8, 9].

Numerous treatments have been documented in the literature, but no consensus exists about the optimal treatment, particularly in advanced stages. Surgery is regarded as the gold standard of treatment. Adjuvants such as chemotherapy, radiotherapy, brachytherapy or intra-arterial superselective chemotherapy have been investigated. Their role, however, is unknown and will require further research [10, 11].

This report details the clinical characteristics, diagnosis, therapy and follow-up of a patient who was diagnosed with advanced squamous cell carcinoma (SCC) of the external ear canal. This is our clinical experience, which also underlines the importance of plastic surgery and adjuvant chemoradiotherapy in managing this disease.

## Case presentation

This patient is a 59-year-old male. Three years ago, the patient was diagnosed with papillary thyroid cancer pT3bN1aM0 and underwent total thyroidectomy and cervical lymphadenectomy, followed by I131 treatment. Additionally, the patient has had persistent otitis media for 5 years and has received many antibiotic rounds. Additionally, the patient had a painless red-pink congenital capillary malformation of 8 × 10 cm in the right parotid region that had been stable for many years. This patient was not followed up with regular routine visits.

The patient was taken to the hospital due to a dull right ear discomfort that worsened gradually over 3 months and was accompanied by purulent discharge from the right ear. The patient was diagnosed with otitis media and given medications, but the infection did not respond well. At the same time, the patient developed a tumour in the external ear canal and was experiencing hearing loss, ear pain and right parotid gland swelling. Clinical examination indicated a large tumour in the right outer ear canal, a darkened surface and warts obstructing the external ear canals. Otolaryngology confirmed the presence of a tumour that covered the whole external ear canal. Histopathological examination of the lesion revealed invasive SCC.

**Figure 1.**
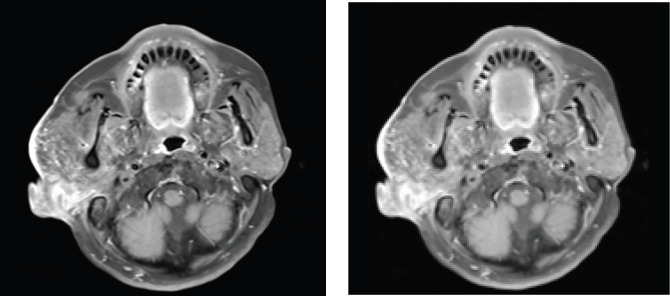
Result of maxillofacial MRI: Right external ear canal obstruction, size 37 × 31 mm, invasion of parotid gland, inner ear canal, with nearby lymph node metastasis.

The results of maxillofacial magnetic resonance imaging (MRI) revealed a 37 × 31 mm blockage of the right external ear canal, decreased signal on T1, elevated signal on T2W and STIR. The tumour infiltrated the right parotid gland, causing it to enlarge and infiltrated the inner ear canal, but did not invade the brain parenchyma. The neighbouring lymph nodes are 18 mm in diameter and are augmented unevenly upon injection (See Figure 1). Positron emission tomography/computed tomography (PET/CT) imaging revealed tumour infiltrating the right parotid tissue. The demarcation is ambiguous. The diameter is 30 mm. 15.7-inch standardized uptake value max (SUVmax). Large lymph node 20 mm in diameter at the right jaw angle enhanced metabolism with SUVmax 13.3. No more abnormalities were discovered. The patient was diagnosed with carcinoma of the right external ear canal (cT4N1M0 according to the Pittsburgh classification).

**Figure 2.**
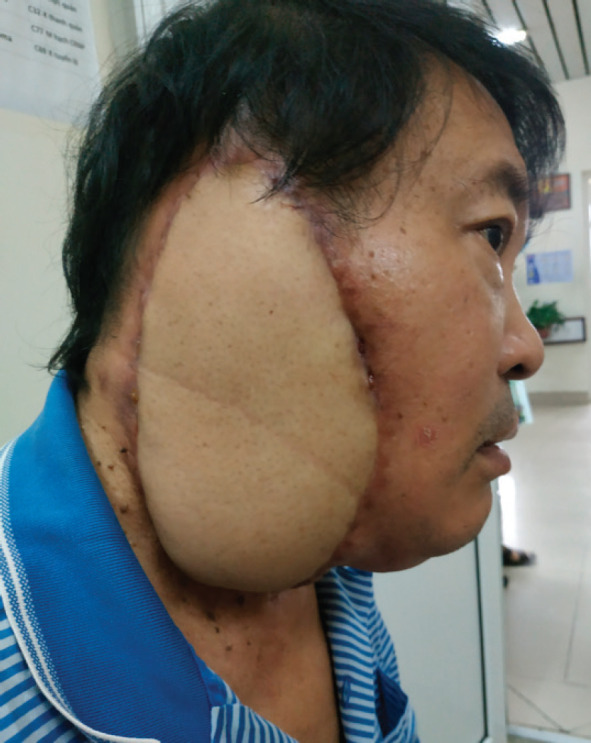
The patient photo after plastic surgery.

The patient underwent significant surgery to remove the external ear canal, middle ear and a portion of the stony bone, as well as the entire right parotid gland, while preserving nerve VII and the right cervical lymph node group I–IV. The patient was then treated with plastic surgery using a free thigh flap (Please refer to Figure 2). The plastic surgery was performed immediately after the tumour resection and regional lymphadenectomy. Postoperative specimen size is 20 × 18 × 5 cm, in which the tumor size is 4.5 × 4 cm. Microscopic examination showed SCC with invasion of nerve fibres, lymphatic infiltration and metastasis to 3/26 lymph nodes. SCC of the right external ear canal pT4N2M0 was diagnosed postoperatively. The postoperative period was stable and chemoradiotherapy was started 8 weeks after surgery. Chemoradiotherapy was used in conjunction with cisplatin on days 1, 22 and 43. The patient received 60 Gy of adjuvant radiation in 30 fractions (volumetric modulated arc therapy (VMAT) technique). The chemoradiotherapy treatment went smoothly. The patient did not interrupt treatment and experienced no serious side effects. The patient is currently stable with no symptoms of recurrence after 28 months of follow-up.

## Discussion

SCC of the external ear canal and temporal bone is a rare malignancy with an incidence of less than 0.2% of head and neck malignancies [12]. Alcohol and cigarette misuse, persistent ear infections and a history of radiation exposure are also common risk factors. Patients frequently present with vague symptoms such as ear discomfort or tinnitus, which can easily be misdiagnosed as chronic ear illnesses such as otitis or chronic mastoiditis. Because the advanced stage of the disease frequently manifests as a large invasive outer ear canal tumour accompanied by the appearance of cervical lymph nodes, it is easy to confuse it with other conditions such as parotid adenocarcinoma, metastatic skin cancer lymph nodes and cervical lymph node metastasis of unknown origin [13].

SCC of the head and neck skin is a type of cancer that develops from the skin’s keratinocytes. The condition is prevalent in white people and frequently manifests clinically as papules, smooth patches, keratosis or ulcers on the skin. SCC of the skin has a modest metastatic rate, accounting for approximately 2%–5% of metastases, most frequently to nearby lymph nodes. In advanced stages, the disease may extend to the external ear canal, parotid gland or metastasise to the cervical or periorbital lymph nodes [13, 14]. Our patient had previously been diagnosed with congenital skin lesions. The patient was examined several times and as the size of the skin lesions has not changed over the years and the surface is smooth, the diagnosis of skin cancer is not considered.

Another situation that needs to be brought up is parotid gland squamous cell cancer. Primary SCC of the parotid gland is rare and needs exclusion of the presence of a primary tumour outside the gland. In this study of 49 patients with SCC parotid, 86% presented with metastatic disease with known primary and only 6% with primary SCCs [15]. Thus, after ruling out other possible causes, the diagnosis of primary SCC of the parotid gland can be made.

Additionally, metastatic SCC metastases from head and neck cancers or other distant primary tumour sites such as the upper gastrointestinal tract, lung, etc., must be checked out. However, no more lesions were discovered in our patient during endoscopy or PET/CT.

**Figure 3.**
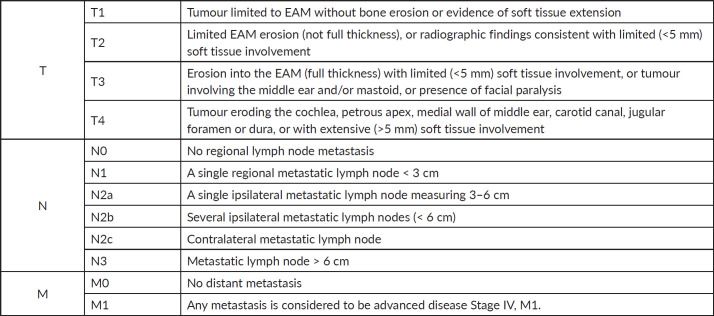
Modified Pittsburgh classification system for carcinoma of the external ear canal.

Due to the disease’s rarity, the AJCC and UICC currently do not have a stage classification for this category of disorders. The most widely used classification system today is the University of Pittsburgh’s TNM system, which was proposed by Arriaga *et al* [6] in 1990 and later modified by Moody *et al* [7] in 2000. Additionally, our patients were staged using this system (See Figure 3).

SCC of the external ear canal is treated in a multimodal way. Aggressive surgery with postoperative adjuvant therapy is usually the treatment of choice for advanced stage disease. The objective is complete surgical excision of the malignant lesion, with negative margins and dissection of the cervical lymph nodes. It is possible to combine this procedure with plastic surgery and adjuvant therapy depending on the postoperative histopathology report. Occasionally, radiotherapy is curative, particularly in the case of early-stage malignancies that are inappropriate for surgery.

For early stage disease, the tumour is still localised to the external ear canal (T1, T2 according to Pittsburgh) and extensive surgical resection of the tumour is the main treatment method. For frail patients with contraindications to surgery, radiotherapy alone is also an effective treatment. A study by Ogawa *et al* [16] in a study of 87 patients with SCC of the external ear canal showed that the 5-year disease-free survival (DFS) rate in patients with stage T1, T2 and T3 was 83%, 45% and 0% in the RT group and 75%, 75% and 46% in the group that received surgery plus radiation therapy. Therefore, the authors suggest that radiotherapy alone may be an alternative to surgery in early stage T1 tumours [16].

For advanced stage tumours, surgery is still the main treatment method. Radiation therapy is used to treat patients with pT3, pT4, positive resection or lymph node metastases following surgery. The role of radiotherapy in adjuvant therapy has also been confirmed in some studies [16, 17]. According to a research by Ogawa *et al* [16], 61% of patients with stage T3, T4 SCC of the outer ear canal treated with surgery and radiation had a 5-year progression-free survival rate of 46%.

One topic to consider is whether the addition of chemotherapy is useful in people with advanced disease. The research is divided on the benefits of chemotherapy in combination with radiation therapy. While Ogawa *et al* [16] shown in a retrospective research that chemicals had no influence in improving DFS [16]. In comparison, Yin *et al* [17] found that combining chemotherapy with surgery and radiation enhanced DFS when compared to the group receiving only surgery and radiation (overall survival (OS) 52.5% versus 28.7%). With this same observation, author Takuya Nagano concluded that concurrent chemoradiotherapy after surgery increases OS and local disease control rates in patients with stage III or IV cancer. The 2-year OS and locoregional control (LRC) rates in patients who had received radiation therapy and concurrent chemotherapy with docetaxel, cisplatin and 5-fluorouracil were 100%. These results were higher than the 2-year OS and LRC rates of 62% and 69%, respectively, in patients who had received radical surgery and radiation therapy [18]. In our patient’s case, the tumour invaded the parotid gland and inner ear canal severely, as well as metastasising to lymph nodes. As a result, the disease has a high recurrence rate and a high rate of local advancement. We chose adjuvant chemoradiotherapy following surgery. VMAT radiation technique was used with a total dose of 60Gy/30frs combined with cisplatin 100 mg/m^2^ on days 1-22-43. The patient tolerated it well during follow-up and treatment, and no associated toxicity was identified.

## Conclusion

External ear canal SCC is a rather uncommon condition. Staging as well as the appropriate treatment plan requires additional research. Pathology, in conjunction with clinical symptoms and imaging, enables a more comprehensive assessment, avoiding confounding factors such as other disorders. Extensive tumour excision in conjunction with plastic surgery followed by adjuvant chemoradiotherapy has demonstrated favourable outcomes and is becoming a more routinely used therapeutic technique.

## List of abbreviations

AJCC, American Joint Committee on Cancer; LRC, Locoregional control; MRC, Magnetic resonance imaging (MRI); OS, Overall survival; PET/CT, Positron emission tomography/computed tomography; SCC, Squamous cell carcinoma; UICC, Union for International Cancer Control; VMAT, Volumetric modulated arc therapy.

## Ethics approval and consent to participate

Not applicable.

## Consent for publication

Written informed consent was obtained from the patients for their anonymised information to be published in this article.

## Availability of data and materials

The datasets used and/or analysed during the current study are available from the corresponding author on reasonable request.

## Conflicts of interest

The authors declare that they have no competing interests.

## Funding

There was no funding for this manuscript.

## Authors’ contributions

The study was designed by Dang Nguyen Van. Material preparation and data collection were performed by Dang Nguyen Van, Thanh Lap Bui and Hoan Pham Van. The data analysis was performed by Minh The Dao and To Ta Van. The first draft of the manuscript was written by Dang Nguyen Van and all authors commented on previous versions of the manuscript. All authors read and approved the final manuscript.
